# Nap‐mediated benefit to implicit information processing across age using an affective priming paradigm

**DOI:** 10.1111/jsr.12728

**Published:** 2018-07-23

**Authors:** Netasha Shaikh, Elizabeth Coulthard

**Affiliations:** ^1^ ReMemBr Group, Institute for Clinical Neurosciences University of Bristol Bristol UK; ^2^ North Bristol NHS Trust Bristol UK

**Keywords:** cognition, healthy function, implicit learning, subliminal

## Abstract

Understanding how sleep‐related information processing affects behaviour may allow targeted cognitive enhancement to improve quality of life. Previous evidence demonstrates that implicitly‐presented cues are processed during subsequent sleep, resulting in enhanced cognition upon waking. We used a masked priming task to investigate this further. To assess sleep‐mediated effects on reactions to implicitly presented primes, participants performed an Affective Priming Task pre‐and‐post 90 min of sleep, compared with an equal period of wakefulness. The Choice Reaction Time Task—a similar binary choice task but without the implicit aspect—was used as a control. Sixteen healthy participants across a range of ages were tested and sleep monitored using electroencephalogram. In stark contrast to the control task, in the Affective Priming Task reaction times significantly improved across all prime types after sleep, but not an equal period of wake. There was no significant change in reaction times on Choice Reaction Time Task after wakefulness or sleep. Rather than a general suppression of all primes, the data are more in keeping with specific strategic optimisation of prime processing during sleep. We plan future work to probe the mechanisms and neuroanatomical substrate of sleep‐mediated prime processing.

## 1 INTRODUCTION

### 1.1 Sleep and cognition: Beyond memory

The case for a link between sleep and cognition is strong, particularly in relation to memory consolidation (i.e. processes such as memorising word lists; Axmacher, Haupt, & Fernandez, 2008; Gais, Lucas, & Born, 2006; Marshall, Helgadottir, & Moelle, 2006). There is good evidence for enhancement of hippocampal‐dependent cognition during sleep (Marshall et al., 2006). However, sleep‐facilitated improvements do seem to be very much task‐ and domain‐dependent, with much left to be uncovered regarding domain‐dissociations and neural underpinnings driving behavioural effects (Mantua, 2018).

Behavioural tasks that measure declarative processes, such as the Number Reduction Task (NRT; Wagner, Gais, & Haider, 2004), indicate sleep‐mediated interpretation of hidden rules (insight), and others highlight abstract problem‐solving within sleep (Beijamini, Pereira, & Cini, 2014; Kirov, Kolev, & Verleger, 2015; Monaghan et al., 2015). Together, this suggests that for some types of cognition, information recall has the potential to not only be strengthened (i.e. memory stabilisation and consolidation), but that information acquired during wakefulness may potentially be processed in some deeper, *qualitative* way during sleep. This may then allow *de novo* behavioural outputs being manifest on awakening, building on what was initially learned (Stickgold & Walker, 2013), for example, greater insight into the information to draw new conclusions. Remarkably, this can occur in the absence of initial intentional, conscious awareness, by processing of implicitly presented cues beneath participants’ conscious awareness (Kirov et al., 2015; Stickgold & Walker, 2013; Wagner et al., 2004).

The picture is much more complex for non‐declarative processes (such as learning new motor skills), with sleep appearing to affect only certain aspects of this (Fischer, Hallschmid, & Elsner, 2002; Walker, Brakefield, & Morgan, 2002). Detailed investigation suggests there may be dissociations between general, explicit skill and implicitly learned aspects of non‐declarative tasks (Csabi et al., 2016; Robertson, Pascual‐Leone, & Press, 2004; Song, Howard, & Howard, 2007).

To explore deeper, qualitative, information processing during sleep, we here decided to utilise a task utilising implicit cues, which, to our knowledge, has not been previously investigated in relation to sleep: the affective priming paradigm (APP). This task was chosen because it removes some of the confounds of previously‐used tasks: for example, while tasks such as the NRT may suggest that implicitly learned information continues to be processed offline, they cannot directly gauge the extent to which the implicit cues have actually been registered by subjects. For instance, with the NRT, only when insight is gained can we be certain that the implicitly presented cue (i.e. a hidden rule within the task) was indeed registered at all. Lack of insight in the NRT may be due to inadequate sleep processing, or it may be caused by variability in the extent implicit cues were processed to begin with, with some participants perhaps not registering implicit cues at all. Although implicit priming and the implicit problem solving occurring in the NRT compared with the APP are due to two different types of processing, the APP nonetheless resolves the ambiguity regarding implicit cues: by looking at objective reaction times to masked prime‐pairs, we can unobtrusively gauge if implicit cues have been processed by the participant.

**Figure 1:**
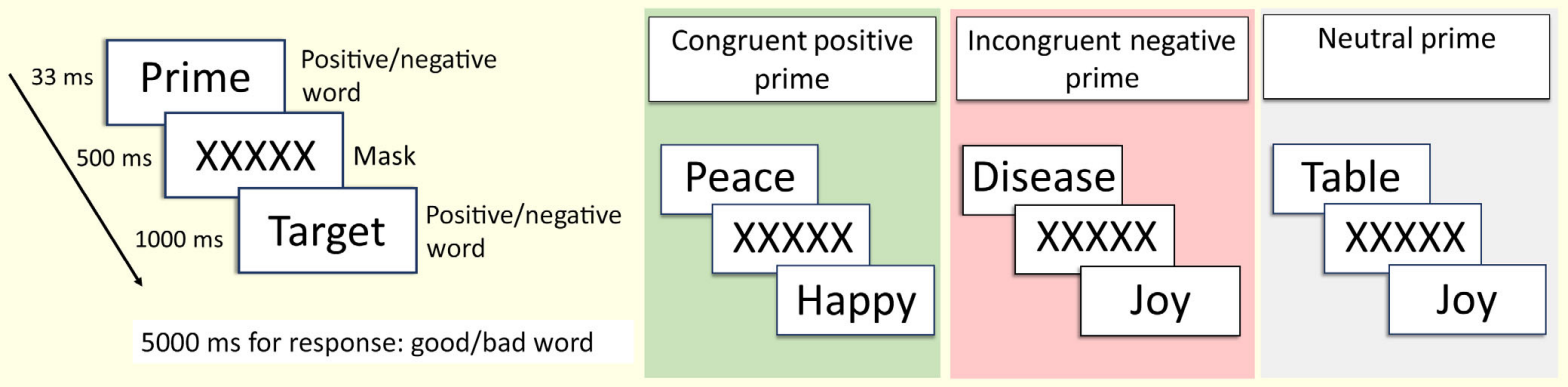
Temporal parameters for each prime‐target pair stimulus in the affective priming paradigm (APP). Note that this does not cover all possible situations: congruent word pairs could be either both positive or both negative; while incongruent word pairs could be a negative prime with a positive target, or a positive prime with a negative target; and neutral prime could be paired with either a positive or negative word

Despite being below the level of conscious awareness, masked priming has been shown to influence subsequent cognitive processing (Boy & Sumner, 2010; Burke, White, & Diaz, 1987; Fazio, Sanbonmatsu, & Powell, 1986). In the APP task used here, stimuli are presented as prime‐target pairs (Figure 1). Participants must make a decision regarding the target word. Unbeknownst to them, a backward‐masked prime briefly appears before the target word, of either the same (congruent) or a different (incongruent) valence as the target stimulus. Implicit stimuli presented in this manner are processed by fast subcortical pathways (Liddell et al., 2005; Morris, Ohman, & Dolan, 1999), below conscious perception (Eimer & Schlaghecken, 2002; Naccache, Blandin, & Dehaene, 2002), activating associated neural responses (Brown, Hagoort, & Chwilla, 2000; Hommel, 1998). If the visible target is congruent with the prime, pre‐activation of conceptually related neural networks generally facilitates and speeds up the response, reducing reaction time (Dell'Acqua & Grainger, 1999), without the subject's conscious awareness (Dehaene et al., 1998; Naccache & Dehaene, 2001). The opposite is true for incongruent prime‐target pairs.

Two distinct stages of processing occur here. The first “priming step” is automatic and implicit: the unseen prime triggers associated subcortical pathways (Brooks et al., 2012; Mendez‐Bertolo et al., 2016; Tamietto & de Gelder, 2010). The next “target response step” requires attention and executive function: after target presentation, if the prime was incongruent, this discrepancy has to be suppressed for the correct decision to be made (Naccache et al., 2002). Responses allow for an unobtrusive, objective measurement of implicit‐cue processing, by measuring interference with behaviour—by primes affecting reaction times.

Critically, the degree to which unconscious primes influence conscious behaviour appears modifiable over time. People are capable of changing their responses to primes, even when stimuli are presented implicitly. Prior studies have presented incongruent primes repeatedly to participants and their responses were modified over time, below participant awareness (Sturmer & Leuthold, 2003; Wolbers et al., 2006), providing evidence that this is potentially an implicitly modifiable type of processing, and not a static one. Based on this potential for below‐awareness modification, we ask whether the masked prime task might be a useful paradigm for exploring cognitive effects of sleep.

Given there is evidence that sleep may foster insight (Wagner et al., 2004; by way of within‐sleep processing of previously presented implicit cues), the primary hypothesis tested here is that sleep modulates processing of implicitly‐presented prime‐cues, resulting in modified behaviour after sleep. That is, in the absence of having to present primes repeatedly to result in a more efficient response, it is possible that within‐sleep processing may elicit the same effect.

There are at least two broad mechanisms through which sleep could affect processing of unconscious primes. If sleep results in a suppression of automatic (i.e. “priming step”) responses to primes across the board, this would be both beneficial as well as detrimental—reaction times to congruent primes would decrease (because of less facilitation being provided by congruence), while reaction times to incongruence would increase (because of less disruption being elicited by incongruency). Conversely, an improvement at the level of error‐detection—an improved ability to choose the correct response and disregard incongruent primes—would result in divergent effects depending on congruence—congruent priming would be left intact, while interfering effects of incongruent priming would be reduced—reflected by an overall improvement in responses.

A napping paradigm was chosen here, given that naps are sufficient to benefit cognition (Korman, Dagan, & Karni, 2015; Lau, Alger, & Fishbein, 2011; Mednick, Nakayama, & Stickgold, 2003) in both younger and older people, making a napping paradigm a robust method of sleep investigation. In addition, comparing the different effects of sleep with an equivalent period of wake is a challenge, as circadian rhythms and consequent hormonal and other effects confound comparisons between day and night (Pan & Rickard, 2015). However, daytime naps provide a window onto a period of sleep matched temporally to a period of wake, albeit that sleep may be of shorter duration and less typical than at night.

To summarise, here we tested the hypothesis that a short bout of sleep facilitates processing of even unconscious information, as manifested by a difference in response to the priming task. We also performed exploratory analysis to see if composition of sleep (e.g. relative percentages of sleep stages) affected the degree of any change.

## 2 MTAERIALS AND METHODS

### 2.1 Ethics

Ethical approval was granted by the Faculty of Health Sciences Research Ethics Committee at the University of Bristol. Participants received an Information Sheet prior to recruitment, and were given the opportunity to ask questions prior to completing a written consent form on their first visit. Participants were offered refreshments and lunch during their visits.

### 2.2 Subjects: Recruitment and screening procedure

Healthy subjects across all ages were recruited via posters and recruitment letters to our research volunteer database. Participants were required to have the capacity to give fully informed consent (as judged by the person screening and Montreal Cognitive Assessment [MoCA] score), be a non‐smoker, and be able to attend four visits to the sleep lab. Exclusion applied if the participant had any diagnosed sleep, neurodegenerative or psychiatric disorder, was taking medication that may interfere with sleep or cognition, or a score of below 26 on the MoCA. These were assessed using the Pittsburgh Sleep Quality Index, Bristol Sleep Profile, Depression Anxiety and Stress Scale (24‐item test), Patient Health Questionnaire (nine‐item test) and questions about their medical history.

Of 63 initial respondents, 16 participants were eligible after screening and able to commit to complete all four testing sessions of the study: 13 female and 3 male across a range of ages (participants were aged between 20 and 91 years, mean age 62 ± 27 years).

### 2.3 Testing protocol

Subjects were instructed not to take caffeinated drinks or alcohol after 18:00 hr the night prior to their visits. Participants completed the St Mary's Sleep Questionnaire to assess their sleep the night before, and the Stanford Sleepiness Scale.

Electroencephalogram (EEG) electrodes were affixed according to the International 10–20 placement system at F3, F4, C3, C4, T3, T4, O1 and O2, referenced to A1 and A2 contralaterally. Two electrooculogram and two chin electrodes were placed to monitor eye movements and muscle activity, respectively, with a ground electrode at the back of the scalp. EEG data were acquired using REMLogic PSG Software (Natus Medical, USA), and recorded using an Embla N700 amplifier (Natus Medical, USA) at a sampling frequency of 256 Hz, high‐pass filter: 0.5 Hz, low‐pass filter: 50 Hz.

Each participant visited the Sleep Lab (at CRICBristol on St Michael's Hill, University of Bristol) on four separate occasions, with each visit at a maximum of 3 weeks after the preceding session.

Sessions were:

1. Habituation Session;
2. Baseline Nap Session;
3. Task with Nap Session^*^;
4. Task with Awake Session^*^.

^*^These sessions were counterbalanced across participants.

Naps commenced between 13:00 hr and 14:30 hr, and lasted for 90 min, to allow for a full sleep cycle. Participant mood was assessed pre‐and‐post sleep session, using the Profile of Mood States scale. The tasks were administered 10 min prior to sleep session and 10 min after waking.

Affective priming paradigm and Choice Reaction Time Task (CRTT) were presented on a Dell laptop with 17ʺ display, using Presentation software (Neurobs, USA) and Cedrus RB‐730 response pad (Cedrus, USA).

### 2.4 APP

The APP (Figure 1) was programmed in Neurobs Presentation software (based in part on Fazio et al., 1986). Incongruent, congruent and neutral prime word trials were presented 40 times each for both “positive” and “negative” prime words; in total, there were 240 trials. The prime in each pair was presented for 33 ms, followed by a mask for 500 ms, followed by target for 1,000 ms. Participants were instructed to indicate whether the target was a “Good” or “Bad” word, and to respond as quickly as possible; 5,000 ms was allowed for a response. The inter‐trial interval was 500 ms post‐response. Trials were presented in blocks of 80, with breaks between blocks.

Words were selected from a validated pool (Bradley & Lang, 1999): words of a similar salience were selected (as validated within the pool of words) to ensure differences were based on valence direction (positive or negative), and not the strength of their valence.

Participants were asked to ensure they were unaware of the prime to confirm that primes remained at an implicit level.

### 2.5 CRTT

Participants were instructed to respond as quickly as possible upon seeing either a blue or red square. Red or blue stimuli were randomly generated. Stimuli were presented for 1,000 ms, 500 ms was allowed for a response and inter‐trial interval was 500 ms post‐response. A total of 150 stimuli were presented during the task (75 blue and 75 red squares, presented at random). The CRTT was administered as a control test, to test for differences pre‐and‐post sleep, as well as between the arbitrary “red or blue” element of the task (compared with APP performance).

### 2.6 Data analysis

Behavioural results were analysed using SPSS software (IBM SPSS Statistics 23.0.0.3) using a repeated‐measures ANOVA, followed by a Bayesian Analysis for attain a Bayes Factor to further analyse main non‐significant results (attained using the online calculator and guidelines at: https://www.lifesci.sussex.ac.uk/home/Zoltan_Dienes/inference/Bayes.htm). EEG data were analysed manually using AASM scoring criteria, on a 30‐s epoch‐by‐epoch basis, in REMLogic; artefacts were manually removed. Resulting sleep stage data were analysed using SPSS.

## 3 RESULTS

### 3.1 Behavioural results

To confirm that primes remained at an implicit level, participants were questioned about the task: some reported being unexpectedly hesitant on some responses but were not aware of what was causing this. All participants were completely unaware of any primes being displayed. The subjective sleep questionnaires revealed no major differences between sessions for duration or sleep satisfaction of prior‐night sleep. Counterbalancing was ensured, with eight participants undergoing the “Task with Nap Session” first and the other eight participants undergoing the “Task with Wake Session” first.

Behavioural data for the APP were analysed using a repeated‐measures ANOVA with within‐subject factors of: (a) testing session (pre‐sleep, post‐sleep, pre‐wake and post‐wake); (b) prime‐target congruency type of the stimulus (congruent, incongruent or neutral); and (c) valence of the prime (negative, positive), with age as a covariate. The assumption of homogeneity of variances was tested and satisfied using Levene's *F*‐test for each outcome variable.

### 3.2 Effects of stimulus

Firstly, looking at the paradigm itself and the differences in reaction times in response to the different stimuli type (i.e. congruent, incongruent and neutral prime‐target pairs), there were significant main effects of prime‐target congruence type (*F*_2,13_ = 30.088, *p* < 0.0005, ηp2 = 0.422).

**Figure 2:**
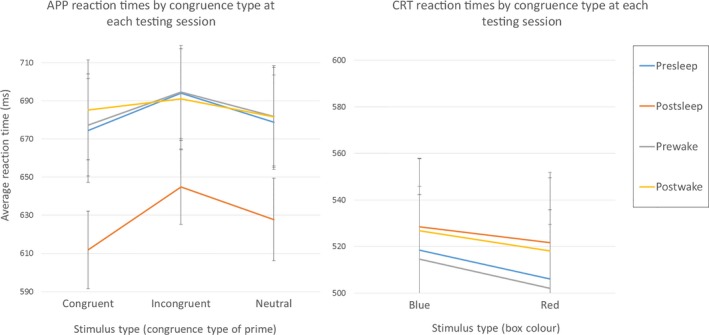
Mean reaction times (±*SEM*) at each testing session (see legend, inset) for (a) Affective Priming Paradigm (APP) and (b) Choice Reaction Time Task (CRTT) for respective stimuli in each task. APP: reaction times to all congruence types improved in the post‐sleep session; CRTT: there were no significant differences between testing sessions

Incongruent prime‐target pairs were associated with the slowest reaction times (Figure 2). Follow‐up pairwise comparisons (with Bonferroni correction) revealed that incongruent pair types (mean = 644.8 ± 17.65 ms) elicited significantly slower reaction times than both congruent pair types (mean = 621.8 ± 18.18 ms; *p* < 0.0001) and neutral prime types (mean = 627.9 ± 18.1 ms; *p* < 0.001). However, reaction times to neutral and congruent pair types did not differ significantly from each other overall (*p* = 0.442). There was no main effect of valence (*F*_1,14_ = 0.156, *p* = 0.699, ηp2 = 0.011).

### 3.3 Effects of testing session (sleep compared with wakefulness)

Reaction times were faster for all congruent, incongruent and neutral prime‐target pair types after a period of sleep, but not after a period of wakefulness (Figure 2).

Mauchly's test indicated that the assumption of sphericity was not met for the testing session variable (*χ* ^2^[5] = 22.1, *p* = 0.001), therefore degrees of freedom were corrected using Greenhouse–Geisser estimates of sphericity (*ε* = 0.64).

Post hoc pairwise comparisons with Bonferroni correction for testing session revealed there was a significant difference (*p* < 0.05) between the pre‐sleep (mean = 643.6 ± 22.1 ms) and post‐sleep testing sessions (mean = 590.9 ± 13.74 ms). There was no significant difference between pre‐wake (mean = 644.7 ± 22.1 ms) and post‐wake (mean = 646.9 ± 22.7 ms; *p* = 0.6). (An estimated Bayes factor of 0.421 was calculated for the pre‐and‐post wake data, suggesting that more testing will be required for further support of the alternative hypothesis.) There were no significant interactions found between testing session and congruency type (*F*_6,9_ = 1.11, *p* = 0.426, ηp2 = 0.426), testing session and valence (*F*_3,12_ = 0.084, *p* = 0.967, ηp2 = 0.021), or testing session and age (*F*_2,13_ = 30.46, *p* = 0.975, ηp2 = 0.017). This indicates that although reaction times were significantly reduced across participants after sleep (i.e. a main effect of testing session, with a significant difference specifically between pre‐and‐post sleep), this sleep‐associated reduction in reaction times did not significantly depend on prime‐target congruence types or valence type.

There was a non‐significant trend for reaction times to congruent stimulus pair types to be improved most after sleep (8.5% reduction), followed by neutral pair types (7.5%). Although responses to incongruent pair types did improve after sleep, the trend suggests that these changed the least (7%). The extent of this post‐sleep difference was similar across ages. Valence did not have a significant effect.

### 3.4 CRTT

The CRTT data were analysed using a repeated‐measures ANOVA with within‐subject factors of: (a) testing session (pre‐sleep, post‐sleep, pre‐wake and post‐wake); (b) type of stimulus (red or blue stimulus) and age were used as covariates. The assumption of homogeneity of variances was tested and satisfied using Levene's *F*‐test for each outcome variable. Mauchly's test of sphericity indicated that the assumption of sphericity was met for all variables.

There was no main effect of stimulus type (*F*_1,14_ = 0.446, *p* = 0.51, ηp2 = 0.031), nor was there an effect of testing session (*F*_3,12_ = 1.47, *p* = 0.27, ηp2 = 0.3), indicating that reaction times did not differ between types of stimuli; nor was there any difference between the pre/post sleep or wake sessions (Figure 2). There were no significant interactions between age and testing session (*F*_3,12_ = 1.76, *p* = 0.208, ηp2 = 0.306), or testing session and stimulus type (*F*_3,12_ = 1.496, *p* = 0.265, ηp2 = 0.272).

### 3.5 Arousal levels

A two‐way repeated‐measures ANOVA of participants’ subjective arousal scores between sessions revealed that scores between sleeping and wakefulness sessions (session type) were not significantly different (*F*_1,14_ = 0.029, *p* = 0.867, ηp2 = 0.002), nor were there significant differences between pre‐and‐post sessions (session timing; *F*_1,14_ = 0.441, *p* = 0.518, ηp2 = 0.031). There were no interactions between session type and timing of the session (*F*_1,14_ = 2.002, *p* = 0.179, ηp2 = 0.125).

### 3.6 Sleep structure and affective priming analysis

A series of multiple linear regression analyses was performed to see if particular sleep stages could predict change in response to the different types of stimuli in the task. Thus, we performed linear regression analysis on change in response time after sleep to either congruent, incongruent or neutral stimuli types, respectively. In each case, predictors were: time spent in N1, N2, N3 or rapid eye movement. Total sleep time (TST) was then analysed separately to avoid issues with collinearity. Regression analyses were performed on all participants’ data. None of the individual sleep stages, or TST, was found to be a significant predictor of performance change after sleep.

## 4 DISCUSSION

### 4.1 Overview of findings

Here, we sought to investigate masked implicit priming to further explore implicit cues and a potential sleep‐mediated response. Reaction times to the priming stimuli were significantly reduced across all pair types after sleep but not wakefulness, and reaction times did not significantly change in the control CRTT after sleep. This combined suggests change in reaction times may be due to a specific effect of implicit information processing occurring during sleep. Therefore, the results here suggest a potentially sleep‐dependent, task‐specific enhancement of brain processing that could optimise human goal‐directed behaviour.

### 4.2 Results of congruence and valence in the APP

As expected, masked primes affected behaviour—overall, reaction times to incongruent primes were significantly slower than congruent or neutral pairs, with a trend for congruent pairs to elicit the fastest responses, indicating the broad behavioural results were as expected for the task. There were no significant effects of valence—demonstrating that congruence type of pairs, rather than their emotional direction, influenced reaction times. This is expected, as selected words were rated of similar salience (Bradley & Lang, 1999; i.e. strength of emotion was the same, despite differing emotional direction).

### 4.3 Limitations and future investigation

One possible confound in this study is that results could potentially be due to general increased arousal after sleep rather than a sleep‐specific optimisation of prime processing. However, this is unlikely, as there was no change in the subjective arousal rating scores in the different sessions, no significant increase in vigilance post‐sleep and no benefit to reaction times on a control task. The CRTT was used to help exclude simple increased arousal effects of sleep; if sleep is benefiting cognition across the board by improved reaction times, then we should expect to see improved performance on both the simple choice task as well as the APP. Improvement solely on the APP, however, may suggest a deeper processing of implicit cues rather than mere increased arousal post‐sleep.

On the basis of our data, we propose that the improvement seen in the APP may be due to implicit processing of cues during sleep. However, to further elucidate within‐sleep processing, future work would have to include the following controls: firstly, a control version of the APP without priming cues, to ensure a binary choice task for more direct between‐task comparison. Further, administering the APP in control condition solely post‐sleep (and not pre‐sleep) would help further determine if a different behavioural response post‐sleep is specifically due to within‐sleep processing, rather than general post‐sleep effects.

Specific sleep stages recorded here did not correlate with post‐sleep performance change; interpretation of this is limited by the relatively small number of participants. Testing further participants across the age range may help draw out task‐ and age‐related differences.

Previous investigation has found that processing of, and responding to, implicitly presented masked primes involves an interplay between fast subcortical pathways and cortical sites (Mendez‐Bertolo et al., 2016; Naccache et al., 2005), with responses being modulated by input from the anterior cingulate cortex by enhancement of the correct response (Brooks et al., 2012; Roelofs, Van Turennout, & Coles, 2006): future investigations may therefore focus on these sites when investigating potential neuroanatomical loci of sleep‐mediated processing (Stickgold & Walker, 2013).

### 4.4 Conclusions

Results suggest that even a short bout of sleep may be sufficient for nuanced implicit cue processing and improved accuracy of response. We propose a putative mechanism where priming cues are processed within sleep, such that the correct response is flexibly enhanced across scenarios; the results here merit further research into this area.

## CONFLICT OF INTEREST

There are no conflicts of interest to declare.

## AUTHOR CONTRIBUTIONS

N.S. was involved in the conception and design of the work, data collection, analysis and interpretation, and drafting and revising the manuscript. E.C. was involved in guiding the conception and design of the work, advice regarding data analysis and interpretation, critical revision of the manuscript and final approval of the version to be published.
