# Supplementary figures and images for: Nap‐mediated benefit to implicit information processing across age using an affective priming paradigm
Source: J Sleep Res. 2018 Jul 23;28(1):e12728. doi: 10.1111/jsr.12728 (PMC7140178; doi:10.1111/jsr.12728)

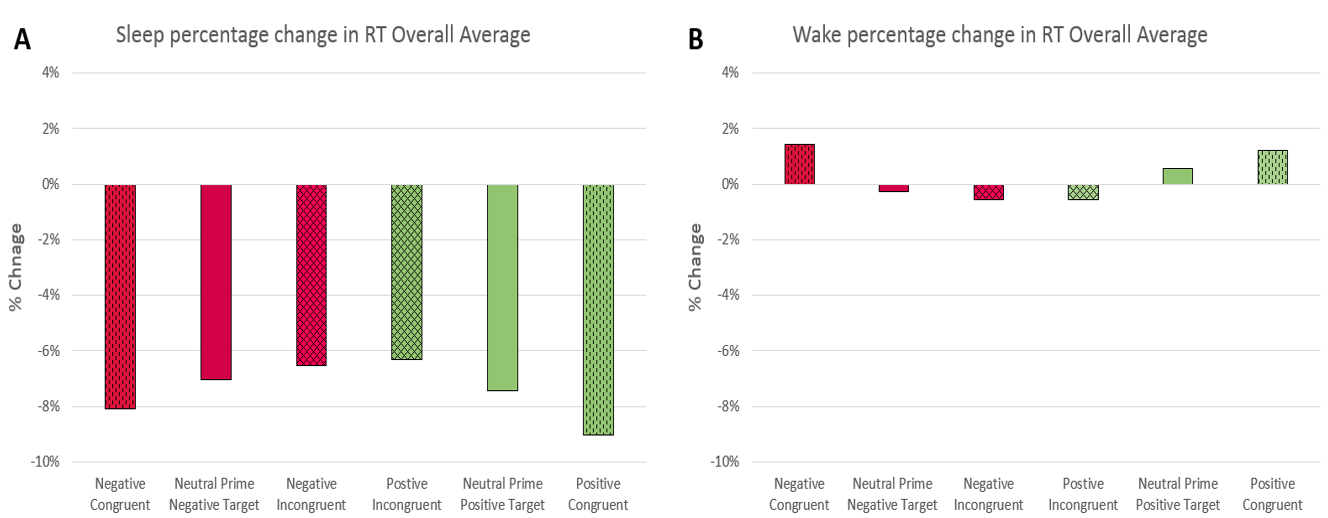

Supplement: Supplementary file 1 [file JSR-28-e12728-s001.tif]
